# Erectile dysfunction and exosome therapy

**DOI:** 10.3389/fendo.2023.1123383

**Published:** 2023-03-09

**Authors:** Huan Feng, Wei Peng, Zhiyao Deng, Jihong Liu, Tao Wang

**Affiliations:** ^1^ Department of Urology, Tongji Hospital, Tongji Medical College, Huazhong University of Science and Technology, Wuhan, Hubei, China; ^2^ Shenzhen Huazhong University of Science and Technology Research Institute, Shenzhen, Guangdong, China

**Keywords:** erectile dysfunction, exosome, extracellular vesicle, diabetes, cavernous nerve injury

## Abstract

Erectile dysfunction (ED), as a common male disease, can seriously reduce the life quality of men and their partners. With the improvement of human living standards, ED is considered to be an important health issue that plagues men. However, it is difficult for existing therapeutic approaches to meet the needs of all patients, so it is necessary to develop novel treatment strategies. Exosomes, as a class of vesicles secreted by cells with bilayer membrane structure, are involved in various physiological and pathological processes in human body and considered to have great therapeutic potentials. This review summarizes the recent advances on exosome therapy with animal models of ED, and proposes the prospect of future research in order to provide a basis for clinical trials and clinical translation.

## Background

More than 5,000 years ago, erectile dysfunction (ED) was mentioned in ancient Egyptian scriptures (1). It was defined until 1993 by the National Institutes of Health (NIH) as the persistent or recurrent failure to attain or maintain a sufficient penile erection for successful penetration (2, 3). ED is a widespread medical issue that seriously affects male health, with 150 million men suffering from it in varying degrees worldwide (4, 5). With males over the age of 40 having a higher prevalence of this issue, it is anticipated that there will be more than 322 million cases of ED worldwide by 2025 (1). In the past, it was frequently believed that psychiatric disorders contribute significantly to the development of ED, but many researches have revealed that organic etiology accounts for more than 80% of ED cases. Cardiovascular diseases, diabetes, dyslipidemia, hypogonadism and nerve damage are independent risk factors for ED (6). More importantly, ED is no longer just related to sexual dysfunction, it may also signal potential vascular endothelial dysfunction, acting as an early indicator of cardiovascular disease (7). Selective phosphodiesterase type 5 inhibitors (PDE5i), like sildenafil citrate, have been widely utilized as the first-line treatment for ED since they enhanced erectile function in 63% of patients and were exploited based on the role of nitric oxide (NO) in cavernous smooth muscle relaxation (8). However, because of the complexity of the pathway that regulates penile erection, up to 35% of patients do not respond to the pharmacological therapy (1). As current treatments do not provide maximum benefits to patients, it is crucial to investigate novel strategies for ED treatments (9).

Extracellular vesicles (EVs) are cell-derived membrane structure with a diameter of 40 nm to 1000 nm. Exosomes and microvesicles are two types of EVs that are released from the endosomal system or shed from plasma membrane, respectively (10). Exosomes are nanosized particles with a diameter of roughly 40-160 nm secreted by various cells under physiological or pathological conditions (11). Exosomes have a bilayer structure that is made by the plasma membrane through encapsulating extracellular components and membrane proteins as well as intersecting with other vesicles and organelles (10, 12). Therefore, exosomes contain many constituents such as metabolites, proteins, lipids and nucleic acids (13, 14). Exosomes are particularly heterogeneous population due to their origin from different cells, as well as the notable differences in their size, composition and effect on the function of recipient cells (15, 16). Exosomes are involved in a variety of physiological and pathological processes in the human body (17), and can be exploited as molecular and signal carriers in intercellular communication (18). Exosome biogenesis enables cells to rapidly and selectively remove proteins from the plasma membrane, facilitating procedures like sperm-egg binding (19). Exosomes are linked to the process of viral infection and are involved in the induction of innate and adaptive immune responses (20). Studies on exosomes and diseases have revealed that exosomes may be involved in cardiovascular and metabolic disorders, take a role in the pathogenesis of neurological disorders, and dynamically affect the growth of cancers (11, 21).

Exosomes are frequently investigated in clinical studies. Exosomal non-coding RNAs (ncRNAs) have been revealed to be expressed differently in most human disorders, which paves the way for their potential application as biomarkers in early stage of diseases (22). Exosomes also serve as drug delivery systems (DDS) for the treatment of cancer (23). For instance, exosomes containing cisplatin inhibited the progression of hepatocellular carcinoma (24). In animal experiments, transplanted bone marrow promoted the regeneration of injured β cell by releasing exosomes enriched with miR‐106b‐5p and miR‐222‐3p (25). Exosomal miRNAs produced by mesenchymal stem cells have been found to exhibit anti-atherosclerotic properties (26). Therefore, it is valuable to further explore the application of exosomes in ED patients, which might be caused by diabetes or cardiovascular disease.

In this review, we introduced the biosynthesis and contents of exosomes, and concentrated on the application of exosomes as well as other types of EVs in the treatment of ED.

## The biogenesis of exosomes

The exact mechanisms of exosome formation are still unclear, but existing studies suggest that the process of exosome biogenesis may be similar in different cell types. Generally, the biogenesis of exosomes is a continuous dynamic process and consists mainly of two invaginations of plasma membrane and the formation of multivesicular bodies (MVBs) containing intraluminal vesicles (ILVs) (11).

First of all, exosomes originate from the inward budding of plasma membrane (that is the first invagination of plasma membrane) to form early sorting endosomes (ESEs) with cell surface proteins and extracellular constituents (27, 28). In some cases, the generated ESEs may be directly merged with the preformed ESEs (11). Afterwards, ESEs either fuse with the plasma membrane for the recycling of sequestered cargoes or convert into late sorting endosomes (LSEs), which can give rise to MVBs with the involvement of endoplasmic reticulum and Golgi complex (29, 30). During the maturation of MVBs, cytoplasmic cargoes including nucleic acids, proteins, lipids, amino acids and metabolites can enter into LSEs by inward budding of endosomal membrane (that is the second invagination of plasma membrane), which leads to the generation of ILVs (27, 29–31). Finally, some MVBs are degraded by the autophagosome or lysosome to maintain cellular homeostasis, while others fuse with the plasma membrane *via* the exocytotic pattern to secrete ILVs, which are also considered as exosomes (32, 33). Notably, exosomes derived from other cells can be taken up by the cells and further fused with ESEs (11).

However, in addition to this canonical model, several studies also revealed the other mechanisms involving in the biogenesis of exosomes. Exosomes can be formed immediately by outward budding through the plasma membrane or delayed released by budding through the deep invagination of plasma membrane (17). Electron microscopy experiments in mesenchymal stem cells and human immune cells have confirmed the existence of the above phenomenon (17, 34–36).

Many factors are associated with the process of exosome biogenesis. Endosomal-sorting complex required for transport (ESCRT) machinery is a key regulator for the conversion of ESEs to LSEs/MVBs, while sometimes this process is independent of ESCRT complex (10, 37). ESCRT machinery is composed of four complexes (ESCRT-0, ESCRT-I, ESCRT-II and ESCRT-III) and the associated proteins (VPS4, VTA1, ALIX and TSG101) (29, 31, 38). ESCRT-0 binds to the ubiquitinated cargoes on the membrane of MVBs followed by the recruitment of ESCRT-I and ESCRT-II thus initiating the invagination of endosomal membrane (10, 39, 40). ESCRT-III is responsible for the scission of ILVs into MVBs (10, 39, 40). Moreover, it is reported that Munc13-4, NEH6, heat shock protein αB-crystallin (HSPB5) and Rab GTPases are required for the endosome maturation and exosome release (41–43).

After exosomes are released by donor cells, they either fuse directly with the plasma membrane of recipient cells to transfer cargoes, or interact with the receptors of recipient cells to activate the corresponding signaling pathways, or undergo endocytosis by recipient cells to release cargoes or fuse with the endosomes to take part in the biogenesis of exosomes in recipient cells (11, 27, 31).

## The contents of exosomes

Exosomes contain not only extracellular substances and plasma membrane proteins, but also a variety of cytoplasmic substances such as DNAs, RNAs, proteins, lipids and metabolites, some of which can be used as biomarkers of exosome (27, 30, 31). The content of exosomes is not constant and varies widely in different cell microenvironments.

Exosomal proteins include: (i) proteins located on the membrane of exosome such as tetraspanins (CD9, CD63, CD81), integrins, MHC class I, II, glycoproteins and other signaling receptors (tumor necrosis factor (TNF) receptor, transferrin receptor), as well as (ii) proteins located in the lumen of exosome such as HSPs (HSP60, HSP70, HSP90), ESCRT machinery (ALIX, TSG101), cytoskeletal proteins (actin, tubulin), enzymes, growth factors and cytokines (27, 44, 45). For instance, transforming growth factor-beta 1 (TGF-β1) and bone morphogenetic protein 2 (BMP2) were detected in plasma exosomes from gastric cancer patients and gastric cancer cell-derived exosomes, respectively (45). These exosomal cytokines were demonstrated to activate the SMAD or PI3K/AKT signaling pathway and give rise to the differentiation of other cells to fibroblasts in gastric cancer (45). Prostate specific membrane antigens (PSMA; PSMA6 and PSMA7) were also identified in blood exosomes from atherosclerosis patients (46). Under the stimulation of lipopolysaccharide macrophage-derived exosomes contain TNF-α and interleukin-1β (IL-1β), which both regulate the inflammatory responses (47).

Exosomal nucleic acids include DNA, mRNA and non-coding RNA (miRNA, lncRNA and circRNA), among which miRNA is one of the most abundant nucleic acids in exosomes. More and more miRNAs contained in exosomes are identified in scientific researches, and they regulate the function of recipient cells or the expression of target mRNA in recipient cells (22, 32). The overexpression of miR-934 in colorectal cancer cell-derived exosomes induced M2 macrophage polarization, activated PI3K/AKT signaling pathway and decreased the PTEN expression (48). High levels of miR-205 were found in circulating exosomes from ovarian cancer patients promoting the angiogenesis and tumor growth (49). Moreover, it was reported that exosomal miRNA from M2 macrophages such as miR-328 promoted the proliferation of pulmonary interstitial fibroblasts and induced the development of lung fibrosis (50). In addition, lipids including cholesterol, ceramides, sphingomyelin and phosphatidylinositol are also contained in exosomes (27).

## The therapeutic potential of exosomes in ED

**Figure 1:**
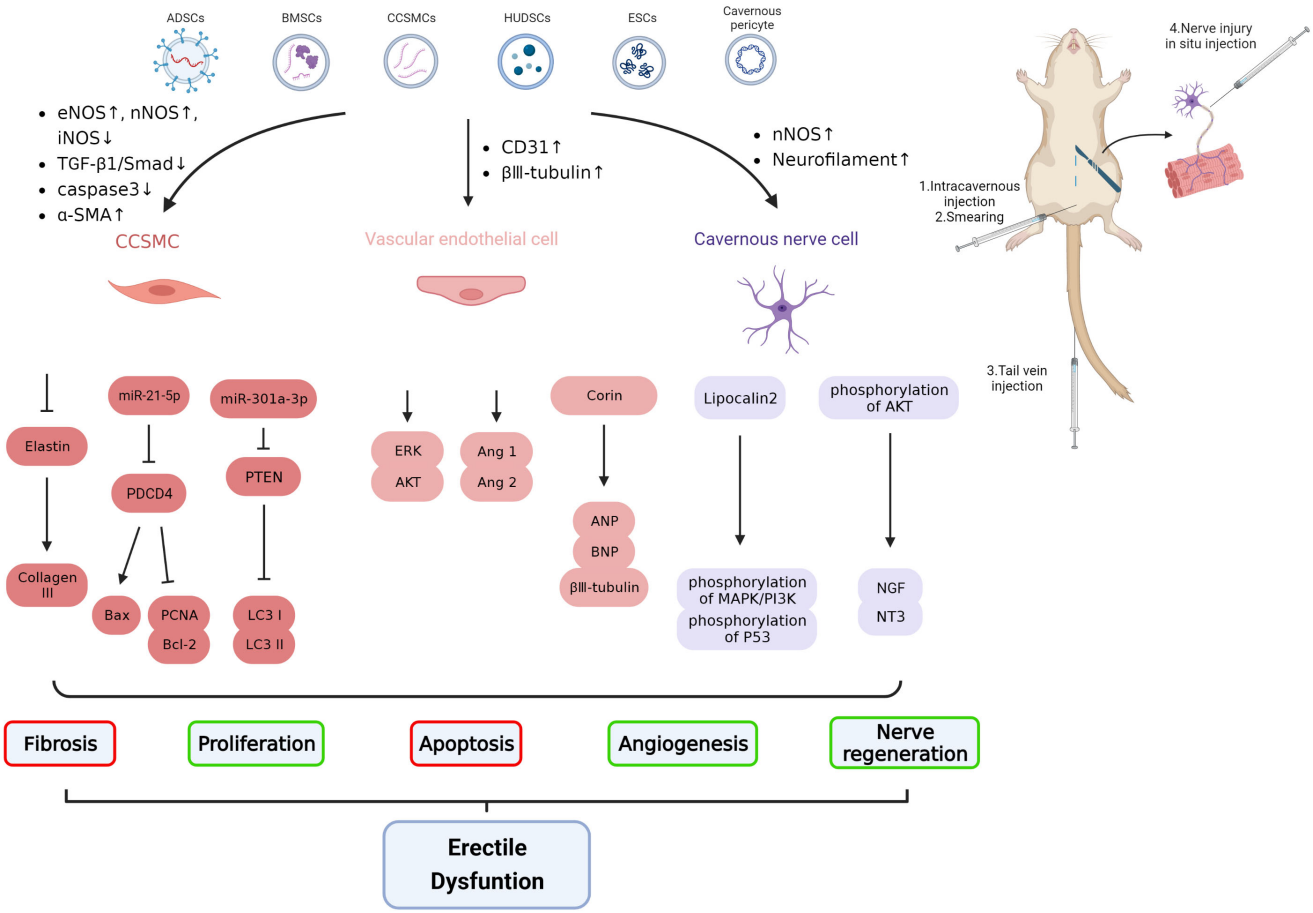
Schematic diagram of the molecular mechanism of exosome therapy for ED. CCSMC, corpora cavernosum smooth muscle cell; eNOS: endothelial nitric oxide synthase; nNOS: neuronal nitric oxide synthase; iNOS: inducible nitric oxide synthase; Ang 1, angiotensin 1; Ang 2, angiotensin 2; ANP: atrial natriuretic peptide; BNP: brain natriuretic peptide; TGF-β1, transforming growth factor-β 1; NT3: neurotrophin 3; NGF, nerve growth factor.

Most studies on exosomes have focused on the field of oncology, while the correlation between exosomes and ED is less studied. The investigation of effects of exosome on the amelioration of ED was analyzed for the first time in 2017 (51). Details of exosome therapy studies in ED models are displayed in **Figure 1** and **Table 1**.

**Table 1:** Therapeutic strategies of exosomes in rat models of ED.

Origin	Model	Modification	Contents of exosome	Validation of exosome	Management of exosome	Indicators of treatment	Outcomes of treatment	Study
**ADSCs**	STZ-induced type II diabetic rats	/	/	TEM; protein markers (CD63, CD81 and calnexin)	Intracavernous injection: 100 μg	4 weeks later ICP/MAP ratio; the expression of Bcl-2, cleaved-caspase 3, CD31 and α-SMA	Prohibiting the apoptosis in cavernous endothelial cells and smooth muscle cells; enhancing erectile function	(51)
**ADSCs**	STZ-induced type I diabetic rats	/	miR-126, miR-130a, miR-132, miR-let7b, miR-let7c	TEM; protein markers (CD9 and CD63)	Intracavernous injection: 0, 10 or 100 μg	4 weeks later The ratio of ICP to MAP, tube formation; the protein expression of endothelial marker vWF and the relative area of smooth muscle to collagen	Increasing endothelial content and angiogenic activity, reversing fibrosis and improving the erectile function in a dose-dependent manner	(52)
**BMSCs**	STZ-induced diabetic rats	miR-21-5p-agomir	miR-21-5p	TEM; protein markers (CD9, CD63 and TSG101)	Tail vein injection: 100 μg	4 weeks later ICP/MAP ratio; apoptosis markers (Bcl-2, Bax, cleaved-caspase 3) and CCSMCs content	Inhibiting expression of PDCD4 and CCSMCs apoptosis; attenuating the erectile dysfunction.	(53)
**ADSCs**	STZ-induced diabetic rats	Corin siRNA	Corin	TEM; protein markers (CD9, CD31, CD63 and CD81)	Intravenous injection: 200 μg	2 weeks later ICP/MAP ratio; the expression of nNOS, ANP, BNP, CD31 and beta-III tubulin; the levels of inflammatory factors (TNF-α, IL-1β, IL-6)	Promoting angiogenesis and nerve content; ameliorating inflammation; improvement of erectile function	(54)
**CCSMCs, ADSCs, BMSCs**	STZ-induced type I diabetic rats	/	/	TEM; protein markers (CD9, CD63, Calnexin and TSG101)	Intracavernous injection: 100 μg	4 weeks later The ratio of ICP/MAP; the expression of nNOS, eNOS and fibrotic markers; the level of NO/cGMP	CCSMC-EXOs have higher peak concentration and longer retention time, restore erectile dysfunction through inhibiting corporal fibrosis and modulating the NO/cGMP pathway	(55)
**ADSCs**	BCNI rats	polydopamine thermosensitive hydrogel	/	TEM; protein markers (CD9 and CD63)	Intracavernous injection: 300 μg	3 weeks later ICP/MAP ratio; the expression of eNOS, nNOS and α-SMA	Healing endothelial cells and neurons; improving the erectile function	(56)
**ADSCs**	BCNI rats	thermo-sensitive hydrogel	/	TEM; protein markers (CD9, CD63 and TSG101)	Nerve injury *in situ* injection: 100 μg	4 weeks later ICP/MAP ratio; the neurite outgrowth; the expression of eNOS and nNOS,	Promoting the repair of nerve injury; improving the erectile function	(57)
**ADSCs, BMSCs**	BCNI rats	/	/	TEM; protein markers (CD63, CD81 and HSP70)	Intracavernous injection: 100 μg	3 weeks later ICP/MAP ratio; the expression of nNOS in DNP and MPG; the level of vWF and the relative area of smooth muscle to collagen	Both alleviating the distortion of normal neural anatomy, smooth muscle atrophy, collagen deposition and erectile dysfunction	(58)
**BMSCs**	BCNI rats	/	/	TEM; protein markers (CD63, Flotillin-1 and TSG101)	Intracavernous injection: 100 μg	4 weeks later ICP/MAP ratio; the expression of nNOS, Caspase-3 and α-SMA; the apoptosis of CCSMCs	Inhibiting apoptosis in CCSMCs; improving the erectile function	(59)
**BMSCs**	Internal iliac artery injury rats	/	/	TEM; protein markers (CD9 and TSG101)	Intracavernous injection: 50 or 100 μg	4 weeks later The ratio of ICP to MAP; the expression of CD31, VEGFA and OCT4; the protein level of eNOS, nNOS, iNOS and SOD; the ratio of smooth muscle to collagen	Promoting cavernous endogenous stem cells to differentiate into cavernous sinus endothelial cells; reducing the oxidative stress damage of corpus cavernosum; improving the erectile function	(60)
**Human urine-derived stem cells**	TGF-β1 induced Peyronie’s disease rats	/	/	TEM; protein markers (CD9, CD63, Alix and TSG101)	Intratunical injection: 100 μg	4 weeks later ICP/MAP ratio; the ratio of smooth muscle to collagen; the expression of α-SMA, TGF-β1 and p-Smad2/3; the gene level of TIMPs and MMPs; the activity of MMPs	Ameliorating the tunica albuginea fibrosis; improving the erectile function	(61)
**ADSCs**	Chronic intermitent hypoxia rats	miR-301a-3p mimics	miR-301a-3p	TEM; protein markers (CD9, CD63 and TSG101)	Intracavernous injection: 400 μg	8 weeks later ICP/MAP ratio; the protein level of eNOS, nNOS and iNOS; the level of apoptosis and autophagy; the ratio of smooth muscle to collagen; the expression of PTEN and TLR4 in DNP	Affecting the apoptosis and autophagy of CCSMCs; improving the erectile function	(62)

ADSC, adipose-derived stem cell; ANP, atrial natriuretic peptide; BCNI, bilateral cavernous nerve injury; BMSC, bone marrow-derived stem cell; BNP, brain natriuretic peptide; CCSMCs, corpus cavernosum smooth muscle cells; cGMP, cyclic guanosine monophosphate; DNP, dorsal nerve of the penis; eNOS, endothelial nitric oxide synthase; ICP, intracavernous pressure; IL, interleukin; iNOS, inducible nitric oxide synthase; MAP, mean arterial pressure; MMP, matrix metalloproteinase; MPG, major pelvic ganglion; MSC, mesenchymal stem cell; nNOS, neuronal nitric oxide synthase; NO, nitric oxide; Smad, contraction of Sma and Mad (Mothers against decapentaplegic); SOD, superoxide dismutase; STZ, streptozotocin; TEM, transmission electron microscopy; TGF, transforming growth factor; TIMP, tissue inhibitor of matrix metalloproteinase; TNF, tumor necrosis factor; TSG101, tumor susceptibility gene 101.

### Diabetic ED

ED is considered as one of the long-term complications of diabetes, which increases the risk of developing ED by 2.5-fold, and more than 50% of people with diabetes are affected by ED (68). Diabetic patients are mainly manifested with hyperglycemia and insulin resistance, usually accompanied by the metabolic syndrome (obesity, hypertension, dyslipidemia), hypogonadism, cardiovascular diseases and neuropathy (69). These manifestations and comorbidities affect the levels of androgen, inflow of arteries, outflow of veins as well as nerve signaling, and ultimately influence the production of reactive oxygen species, NO, cyclic guanosine monophosphate (cGMP) and nitric oxide synthase (NOS), and the function of endothelial cells and corpus cavernosum smooth muscle cells (CCSMCs) during the erection of penis (69, 70).

Chen et al. established a rat model of type II diabetes and isolated exosomes from adipose-derived stem cells (ADSCs) by ultracentrifugation (51). They found that ADSC-derived exosomes promoted the recovery of erectile function by increasing the ratio of maximal intracavernous pressure (ICP) to mean arterial pressure (MAP), elevating the endothelium and smooth muscle contents and decreasing the apoptosis in cavernous endothelial cells and smooth muscle cells (51). In addition to type 2 diabetes, ED was also induced following type 1 diabetes. Zhu et al. injected type 1 diabetic ED rats with three different doses of ADSC-derived exosomes (0, 10 or 100 μg, respectively) (52). It was demonstrated that exosomes improved the erectile function by reversing fibrosis, increasing endothelial content and angiogenic activity in a dose-dependent manner. Interestingly, they also performed miRNA sequencing on the extracted exosomes and found that some functional miRNAs were contained in these exosomes, including proangiogenic miRNAs (miR-126, miR-130a, miRNA-132) and antifibrotic miRNAs (miR-let7b and miR-let7c). Although the role of these miRNAs in exosomes in the treatment of ED has not been validated *in vitro* or *vivo*, these findings revealed the potential mechanisms of exosome therapy in ED, which opened up a new perspective for the future investigation of exosomes in the treatment of ED.

Notably, donor cells can be modified by transgenic methods so that exosomes released by these cells can contain a large number of specific cargoes. For example, bone marrow-derived stem cells (BMSCs) treated with miR-21-5p-agomir resulted in the increase of miR-21-5p in BMSC-derived exosomes, which decreased the expression of target gene programmed cell death 4 (PDCD4) in CCSMCs, attenuating the erectile dysfunction through leading to the proliferation and apoptosis inhibition of these cells (53). Meanwhile, exosomes derived from ADSCs transfected with corin siRNA promoted the neurovascular function and suppressed the levels of inflammatory factors including TNF-α, IL-1β and IL-6 (54). Our group also investigated the therapeutic effects of exosomes in ED. Distinct from the above study, we isolated exosomes from CCSMCs and found that this type of exosomes was more easily retained in the corpus cavernosum and better ameliorated the diabetes-induced erectile dysfunction compared with exosomes from ADSCs and BMSCs (55).

### Bilateral cavernous nerve injury-induced ED

Prostate cancer surgery and other pelvic surgeries often result in damages to the cavernous nerve, which originates from the major pelvic ganglion (MPG) and controls the relaxation and contraction of CCSMCs, thus regulating the erection of the penis (71). It was reported that up to 80% of prostate cancer patients suffer from ED after radical prostatectomy (72). Many molecules and signaling pathways contribute to the development of ED during the injury of cavernous nerve, for instance TGF-β, hydrogen sulfide, NO pathway, RhoA/ROCK pathway or oxidative stress-related pathway (73, 74). The dysregulation of these factors after BCNI leads to the tissue fibrosis, as well as phenotypic transformation and apoptosis of CCSMCs (73).

Exosomes isolated from ADSCs and BMSCs have been applied not only to diabetic ED but also to BCNI-induced ED. Ouyang et al. found that four weeks after injection with exosomes into the corpus cavernosum of BCNI-induced ED rats, the erectile function was obviously improved *via* inhibiting apoptosis in CCSMCs (59). Similarly, ADSC-derived and BMSC-derived exosomes have been shown to be effective in recovering erectile dysfunction in BCNI rat model (58). Both of them can alleviate the distortion of normal neural anatomy, smooth muscle atrophy and collagen deposition, which impaired the erection of penis.

In order to enhance the uptake of exosomes and the efficacy of treatment, some research teams have developed specific hydrogels and mixed them with exosomes for the treatment of ED. Liang et al. fabricated polydopamine thermosensitive hydrogels, which exhibited sol-gel transition at body temperature and allowed exosomes to be released slowly within two weeks (56). *In vivo* experiments confirmed that ADSC-derived exosomes loaded within polydopamine thermosensitive hydrogel improved the erectile function by healing the endothelial cells and neurons in penis. On the other side, ADSC-derived exosomes encapsulated into the thermosensitive hydrogel significantly repaired the cavernous nerves injury in rats, thus restoring erectile function (57).

In addition, a study showed that Schwann cell-derived exosomes promoted nerve regeneration of MPG and cavernous nerve with MPG, suggesting that they may provide potential therapeutic options for ED treatment (75). Since this study only involved ex vivo experiments, appropriate *in vivo* experiments are needed to further confirm the findings.

### Other types of ED

So far, exosomes have been introduced to treat some other types of ED in addition to diabetic ED and BCNI-induced ED. Vascular ED accounts for a large proportion in elderly patients with ED, which can be caused by atherosclerosis, trauma and surgery. It has been shown that in internal iliac artery injury-induced ED rats, BMSC-derived exosomes promoted cavernous endogenous stem cells to differentiate into cavernous sinus endothelial cells while effectively reduced the oxidative stress damage of corpus cavernosum (60). These findings provided a novel insight and strategy for the clinical treatment of severe arterial injury ED.

Since erectile dysfunction is often observed in patients with obstructive sleep apnea, the researchers developed a rat model of chronic obstructive hypoxia-induced ED. The miR-301a-3p-enriched ADSC-derived exosomes affected the apoptosis and autophagy of CCSMCs by targeting PTEN and TLR4, and ultimately improved erectile function (62). Furthermore, Yang et al. demonstrated that human urine-derived stem cell (HUSC)-derived exosomes ameliorated the fibrosis in tunica albuginea and restored erectile function in Peyronie’s disease rats (61).

## The therapeutic potential of other subsets of EVs in ED

Due to the low production of EVs, Kwon et al. developed embryonic stem cell (ESC)-derived EV-mimetic nanovesicles (ESC-NVs), and diabetic mice were received intravenous injection of 0.1, 0.5, 1, 2 or 5 μg ESC-NVs for 2 times, respectively. The results revealed that those ESC-NVs enhanced penile neurovascular regeneration by boosting the expression of angiogenic and neurotrophic factors (63). A team of researchers from South Korea extracted EV-NVs from cavernous pericytes, and they found that these NVs can significantly promote neurovascular regeneration and ameliorate erectile dysfunction in both diabetic and BCNI-induced ED rats (64, 65). The studies also compared the efficacy of different doses of NVs and found that the higher the dose, the better the recovery of pathological changes and erectile dysfunction. Moreover, EVs derived from HUSC (HUSC-EVs) have been proven to ameliorate erectile dysfunction effectively (66, 67). In contrast to the conventional intracavernous or intravenous injection, Zhuang et al. mixed HUSC-EVs with hyaluronic acid and smeared them on the glans of the rats several times (67). The results showed that this administration can also improve apoptosis, angiogenesis, and smooth muscle regeneration as the conventional injection **Table 2**.

**Table 2:** Therapeutic strategies of other subsets of EVs in rat models of ED.

Origin	Model	Modification	Contents of EVs	Validation of EVs	Management of EVs	Indicators of treatment	Outcomes of treatment	Study
**ESCs**	STZ-induced diabetic mice	/	/	TEM; protein markers (CD63, CD81, GM130 and TSG101)	Intracavernous injection: 0.1, 0.5, 1, 2 or 5 μg for 2 times	2 weeks later The ratio of total ICP or maximum ICP to MSBP; the content of cavernous pericyte, smooth muscle cell and endothelial cell; the level of tube formation and aortic ring micro-vessel outgrowth; the expression of angiogenenic and neurotrophic factors	Enhancing penile neurovascular regeneration; ameliorating erectile dysfunction	(63)
**Cavernous pericyte**	CNI rats	/	/	TEM; protein markers (CD81, GM130, Alix and TSG101)	Intracavernous injection: 0.2, 1 or 5 μg	2 weeks later ICP/MSBP ratio; the content of cavernous pericyte and endothelial cell; the content of nNOS and neurofilament in DNB; the level of tube formation; the expression of neural and neurovascular regeneration markers	Promoting neurovascular regeneration; improving erectile dysfunction	(64)
**Cavernous pericyte**	STZ-induced diabetic mice	/	/	TEM; protein markers (CD81, GM130, Alix100 and TSG101)	Intracavernous injection: 0.5, 1 or 5 μg	2 weeks later The ratio of total ICP or maximum ICP to MSBP; the expression of PECAM-1 and α-SMA; the level of tube formation and aortic ring micro-vessel outgrowth; the expression of neural and neurovascular regeneration markers	Promoting penile angiogenesis and neural regeneration; improving erectile dysfunction	(65)
**Human urine-derived stem cells**	STZ-induced diabetic rats	/	miR-21-5p, let-7 family, miR-10 family, miR-30 family, miR-148a-3p	TEM; protein markers (CD63 and Calnexin)	Intracavernous injection: 100 μg	4 weeks later ICP/MAP ratio; the expression of nNOS and eNOS; the relative area of smooth muscle to collagen	Alleviating the fibrosis; attenuating the erectile dysfunction.	(66)
**Human urine-derived stem cells**	STZ-induced type II diabetic rats	hyaluronic acid	/	TEM; protein markers (CD9, CD63, TSG101 and Calnexin)	Smearing on the glans: 2 × 10^9^ particles for 5 or 10 times	4 weeks later ICP/MAP ratio; the formation of HUVECs capillary-like Structure; the gene level of Bcl-2, Bax and SOD2; the expression of nNOS, eNOS and iNOS; the relative area of smooth muscle to collagen	Improving apoptosis, angiogenesis, and smooth muscle regeneration; enhancing erectile function	(67)

CNI, cavernous nerves injury; DNB, dorsal nerve bundle; eNOS, endothelial nitric oxide synthase; ESC, embryonic stem cell; HUVEC, human umbilical vein endothelial cell; ICP, intracavernous pressure; iNOS, inducible nitric oxide synthase; MAP, mean arterial pressure; MSBP, mean systolic blood pressure; nNOS, neuronal nitric oxide synthase; SOD2, superoxide dismutase 2; STZ, streptozotocin; TEM, transmission electron microscopy; TSG101, tumor susceptibility gene 101.

## Future prospects

Basic and preclinical researches have shown that exosomes have the potential to treat ED. Since the research in this field is still at a preliminary stage, there are no ongoing or completed clinical trials. Although exosomes exhibit good therapeutic effects in rat and mouse, rodent models are insufficient to predict human clinical outcomes. Exosome therapy in ED needs to be validated in higher-order animals that more closely mimic the physiological and clinical characteristics of the human body. In fact, a large number of clinical trials based on exosomes have been completed in many other diseases (76–83), so we need to speed up the pace to more comprehensively confirm the therapeutic effect of exosomes on ED and promote the translation from animal studies to clinical trials. In addition, it is worth noting that most of the existing studies only focus on the therapeutic effects of exosomes on ED and related mechanisms, but there are relatively few explorations of exosome contents. In the future, we should take actions to further understand the role of key molecules identified in exosomes in the treatments of ED.

There are still several key issues to be resolved, including long-term safety, optimal source of exosomes, optimal therapeutic method, dose and course, appropriate delivery system and elucidation of the specific mechanism. Most of the studies used exosomes derived from allogeneic cells to treat ED through intracavernous injection. Tail vein injection, intratunical injection and nerve injury *in situ* injection are also working with exosomes during the treatment of ED, although there are only a few reports in the literature. Considering the low yield of human exosomes, how to obtain sufficient exosomes for future clinical use is worth thinking. The sources of exosomes and therapeutic doses need recognized standards, and the comparison of the effects of exosomes from different sources requires more rigorous evidence to support it. Meantime, the doses of exosomes in previous studies vary greatly, which makes the appropriate dose necessary. Most researchers have injected exosomes at a dose of 100 μg and achieved good results, but this only applies to rodent models. Despite the fact that exosomes do not express MHC proteins and will not cause immune tolerance and cell malignancies (84), it is still unknown whether there will be other adverse advents in the further research owing to the fact that the contents of exosomes have not been fully clarified. While exosomes may not be able to completely reverse the pathological changes in the corpus cavernosum, they can delay the progression of the disease, which is enough to improve sexual function and improve the quality of life. Due to the complexity of ED pathophysiology, combination therapy may be more effective, such as oral medications and physical therapy.

Moreover, exosomes have some applicative advantages. First, exosomes can be stored for a long time at low temperature, which is convenient for storage and transportation (84). Secondly, the protein, nucleic acid and other contents in exosomes are encapsulated by lipids, which is the structural basis for good stability (85). It is important to note that exosomes may circumvent many issues related to ethics. The exosome may be the future of ED treatment, nevertheless research is still at the preliminary stage. Basic researches and clinical trials of exosomes in the treatment of ED lay a solid foundation for the clinical translation of exosome therapy.

## Author contributions

HF: Conception, methodology, data investigation & manuscript draft. WP: Conception, data investigation & manuscript draft. ZD: Conception, data investigation & manuscript draft. JL: Manuscript reviewing & project supervision. TW: Manuscript reviewing & project supervision. All authors contributed to the article and approved the submitted version.
